# Acute Heat Exposure Alters Autophagy Signaling in C2C12 Myotubes

**DOI:** 10.3389/fphys.2019.01521

**Published:** 2020-01-08

**Authors:** Corey M. Summers, Rudy J. Valentine

**Affiliations:** ^1^Department of Kinesiology, Iowa State University, Ames, IA, United States; ^2^Immunobiology Graduate Program, Iowa State University, Ames, IA, United States

**Keywords:** heat, AMPK, autophagy, LC3, skeletal muscle

## Abstract

Autophagy is a major intracellular degradation process that is essential for the clearance of unnecessary proteins/organelles and the maintenance of cellular homeostasis. The inhibition of autophagy results in cellular consequences associated with many skeletal muscle pathologies, and therapies designed to elevate autophagic activity may provide protection from such pathologies. Acute exposure to low levels of heat has therapeutic effects; however, the impact of heat on skeletal muscle autophagy remains unclear. In the present study, C2C12 myotubes were maintained at 37°C thermoneutral (TN) or heated at 40°C heat treatment (HT) for 1 h. Myotubes were harvested immediately after heating, or returned to 37°C for recovery of 2 or 24 h. HT resulted in an elevation in pAMPK (T172), Beclin-1, and LC3 II, a marker for autophagosome formation, but no change in p62. In the context of autophagy inhibition with Bafilomycin A1, HT resulted in lower LC3 II compared to TN. The applied heat load induced the heat shock response, as evidenced by immediate upregulation of HSF1 and Hsp70. Hsp70 continued to increase during recovery, whereas pHsp27 was downregulated acutely in response to HT, but retuned to TN levels by 2 h of recovery. HT also reduced the phosphorylation of the MAP-kinases p38 and JNK. These findings suggest that an acute, short bout of mild heat may be beneficial to skeletal muscle by increasing AMPK activity, markers of autophagasome formation, and the heat shock response.

## Introduction

Macroautophagy, herein referred to as autophagy, is an essential and highly regulated mechanism for the bulk degradation of cytosolic proteins and organelles in mammalian cells. While the basal levels of autophagy might be tissue- or cell type-dependent, the induction of autophagy by stress is highly conserved (Mizushima and Komatsu, 2011). Hypoxia, oxidative stress, nutrient depletion, and exercise are just some of the many stressors that have been shown to increase autophagic activity (Vainshtein and Hood, 2016). This increase in autophagy serves as an adaptive response mechanism, essential for the cell’s survival and return to homeostasis.

Autophagy has been shown to be critical for skeletal muscle homeostasis (Masiero et al., 2009; Grumati et al., 2010). A deficiency in autophagic flux results in an accumulation of aggregated proteins and damaged organelles, leading to skeletal muscle atrophy, and weakness (Masiero et al., 2009; Kim et al., 2013). Several myopathies, including muscular dystrophy and glycogen storage disease have an abnormal reduction in autophagy that contributes to disease progression (Malicdan et al., 2008). Similarly, skeletal muscle autophagy is dysregulated in metabolic diseases, including insulin resistant and diabetes (Hotamisligil, 2010; He et al., 2012; Møller et al., 2017). Importantly, in some models, structure and function of skeletal muscle can be restored by pharmacological reactivation of autophagy (De Palma et al., 2012, 2014; Pauly et al., 2012). Thus, strategies to increase skeletal muscle autophagic flux may have important implications in the context of muscle myopathies, obesity, insulin resistance, and aging, among others.

The contraction of skeletal muscle generates a large amount of physical and metabolic stress, which instigates high levels of protein turnover (Röckl et al., 2007; Poortmans et al., 2012). Muscular contraction during exercise induces a state of metabolic stress in skeletal muscle, decreasing ATP content, altering molecular messengers, and inducing protein and organelle damage (Röckl et al., 2007), and upregulation of autophagy (Sandri, 2010; He et al., 2012). This increase in autophagy is essential for exercise-induced benefits in glucose homeostasis (He et al., 2012), and likely other homeostatic processes. During exercise, skeletal muscle temperature increases by several degrees (Brooks et al., 1971; Saltin et al., 1972), which appears to account for at least some of the biochemical responses to exercise. Exposure to short bouts of heat, mimicking temperatures achieved during exercise, recapitulates many of the beneficial effects exercise, including protection against muscle atrophy (Naito et al., 2000; Selsby and Dodd, 2005), and improvements in glucose homeostasis (Hooper, 1999; Gupte et al., 2009, 2011) and vascular function (Brunt et al., 2016a, b). and may be an important component of the beneficial effects of exercise. While there is literature supporting the adverse effects of prolonged heating on skeletal muscle autophagy (Brownstein et al., 2017), less is known about the effects of acute heating on autophagy signaling in skeletal muscle.

The purpose of the present study was to investigate the effects of an acute, short bout of HT on autophagic signaling in skeletal muscle cells. To do this, C2C12 myotubes were exposed to 1 h of HT at 40°C, with and without recovery. We hypothesized that a single short bout of HT would increase autophagic signaling and the heat shock response in skeletal muscle cells.

## Materials and Methods

### Materials

C2C12 myoblasts (CRL-1772) were purchased from ATCC (Manassas, VA, United States). Dulbecco’s Modified Eagle’s medium (DMEM), Penicillin-Streptomycin (P/S), fetal bovine serum (FBS), and horse serum (HS) were purchased from Invitrogen (Grand Island, NY, United States). L-Glutamine was purchased from Thermo Fisher Scientific (Carlsbad, CA, United States). Bafilomycin A1 (BafA1) was purchased from Tocris Bioscience (Minneapolis, MN, United States).

### Cell Culture

C2C12 myoblasts were cultured at 37°C, with 5% CO_2_, in complete growth medium, containing normal glucose (5.5 mM) DMEM supplemented with 10% FBS, 1% L-Glutamine, and 1% P/S. Growth media was replenished every 24–48 h and cells were passaged upon reaching 70–90% confluency. Once at 70–90% confluency, myoblasts were differentiated into myotubes by the replacement of growth media with differentiation media, containing normal glucose (5.5 mM) DMEM supplemented with 2% HS and 1% P/S. Differentiation was performed for 5 days, with the media being replenished every 24–48 h (Supplementary Figure S1). On day 5 of differentiation, medium was changed to serum-free medium, containing normal glucose (5.5 mM) DMEM supplemented with 1% P/S.

### Treatment of Cells

**FIGURE 1:**
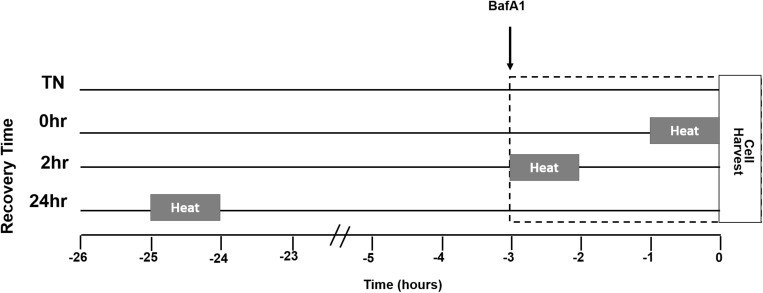
Myotube heat treatment (HT) experimental design. Myotubes were placed into one of four groups, thermoneutral (TN), 1 h of HT with no recovery (0 h), 1 h HT with 2 h of recovery (2 h), or 1 h HT with 24 h of recovery (24 h). Heating at 40°C began 1 h prior to the amount of recovery time designated for the group. Bafilomycin A1 (BafA1) was added 3 h prior to cell harvest for each group. The lines next to the group name represent time spent in 37°C, from the start of the experiment. The gray bars represent time spent in 40°C in relation to BafA1 and cell harvest. The dotted box indicates duration of BafA1 treatment.

A representation of the study design is given for heat treatment (HT), BafA1 treatment, and recovery times (Figure 1). Myotubes were either maintained at 37°C or given a single bout of 40°C HT for 1 h, by placing the cultures into an incubator at 40°C, with recovery times of 0, 2, or 24 h at 37°C. To gain insight into autophagic flux, some cells were treated with the autophagy inhibitor BafA1 (100 nM) 3 h prior to cell harvesting. Three hours was selected, per recommendation, and to standardize the length of time autophagy was inhibited across all treatments. Given the differences in recovery time, adding BafA1 prior to heating would lead to large differences in the length of BafA1 exposure, and the longer durations would likely impact cellular health (Klionsky et al., 2016).

### Cell Lysate Preparation

Upon completion of the treatment protocol, the serum-free media was removed, and cells were washed twice on ice with phosphate buffered saline (PBS). Cells were then lysed with 1X cell lysis buffer (Cell Signaling; Danvers, MA, United States) (20 mM Tris–HCl (pH 7.5), 150 mM NaCl, 1 mM Na_2_EDTA, 1 mM EGTA, 1% Triton, 2.5 mM sodium pyrophosphate, 1 mM beta-glycerophosphate, 1 mM Na_3_VO_4_, 1 μg/ml leupeptin), containing 1X Halt Protease Inhibitor Cocktail (Thermo Fisher Scientific, Waltham, MA, United States) and 1X Phosphatase Inhibitor Cocktail 3 (MilliporeSigma; Burlington, MA, United States), and removed from wells using a sterile, double-ended cell lifter (CellTreat Scientific Products; Pepperell, MA, United States). Cellular debris was removed from lysates by centrifugation at ∼20,000*g* for 15 min at 4°C, and the supernatant was removed and stored at −80°C until analysis. Protein concentrations were assessed using the Pierce BCA Protein Assay Kit (Thermo Fisher Scientific, Waltham, MA, United States).

### Western Blot Analysis

Samples were then prepared for western blot analysis by denaturation in Laemmli sample buffer (Bio-Rad; Hercules, CA, United States), containing the reducing agent dithiothreitol (DTT), at 95°C for 5–10 min. The prepared samples were equally loaded into 4–15%, 4–20%, or 8–16% Stain-Free Criterion TGX gels (Bio-Rad) to final protein amounts of 15–30 μg per lane. After electrophoresis was complete, proteins were transferred to PVDF membranes (MilliporeSigma). To ensure proper electrophoresis and transfer of proteins, as well as obtain a precise measurement of total lane protein, membranes were subjected to Bio-Rad’s Stain-Free protocol to obtain an image of total lane protein (Ghosh et al., 2014). Protein-coated membranes were then blocked, in 5% fat-free milk in Tris–buffered saline containing 0.1% Tween-20 (TBS-T), for 1 h at room temperature. After blocking, membranes were cut and probed with primary antibodies (1°) overnight at 4°C. Following 1° incubation, membranes were probed with HRP-linked anti-rabbit or anti-mouse secondary antibodies (Cell Signaling), at a concentration of 1:2000–1:5000, for 1 h at room temperature. Western blot images were captured with the ChemiDoc^TM^ XRS Imaging System (Bio-Rad), and images were analyzed using Image Lab^TM^ 6.0 Software (Bio-Rad). All bands of interest were normalized to total protein. The primary antibodies specific for AMPKα (#2532), phospho-AMPKα^T172^ (#2531), Atg3 (#3415), Atg16L1 (#8089), IκBα (#4814), phospho-JNK^T183/Y185^ (#4668), LC3A/B (#12741), mTOR (#2983), phospho-mTOR^S2448^ (#2971), phospho-NFκB^S536^ (#3033), PI3K Class III (#4623), p38 (#9212), phospho-p38^T180/Y182^ (#9211), phospho-ULK1^S757^ (#6888), and phospho-ULK1^S555^ (#5869), as well as the secondary horseradish peroxidase (HRP)-linked antibodies (#7074, #7076) were purchased from Cell Signaling Technology. Antibodies specific for Beclin-1 (sc-48341), p62 (sc-28359), HSF1 (sc-17757), Hsp27 (sc-13132), phospho-Hsp27^S82^ (sc-166693), and Hsp70 (sc-24) were obtained from Santa Cruz Biotechnology, Inc. (Dallas, TX, United States).

### Statistics

Data are presented as mean ± SEM from 3 to 5 independent experiments, with 2–3 technical replicates each. Statistical differences between groups were analyzed using a One-Way ANOVA, however, a Two-Way ANOVA was used when comparing heat and bafilomycin A1 treatments. When appropriate, this was followed by a *post hoc* comparison using the Tukey test. Statistical significance was set to *p* < 0.05.

## Results

### A Single Bout of 1 h 40°C Heat Treatment (HT) Induces AMPK Signaling

**FIGURE 2:**
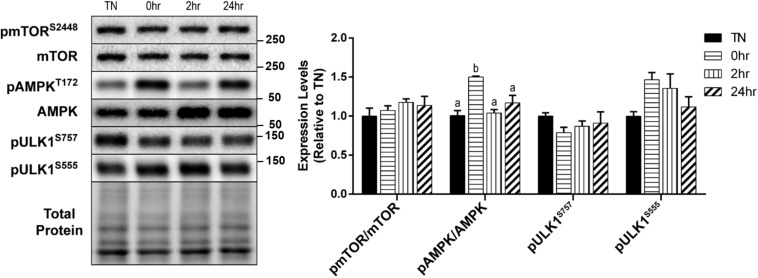
Assessment of skeletal muscle autophagy initiation signaling in response to 1 h 40°C HT. C2C12 myotubes were left at 37°C, TN, or exposed to 40°C for 1 h, HT, with cell harvest occurring immediately 0 h, 2 h, or 24 h post-heating. Protein lysates of TN and heat treated myotubes were analyzed by western blot for autophagy initiation proteins: pmTOR^S2448^, pAMPK^T172^, pULK1^S757^, and pULK1^S555^. Western blots and the quantifications are shown for all proteins. Bars that do not share similar letters denote statistical significance, *p* < 0.05 using a one-way ANOVA. Values are presented as mean ± SEM, compared to TN.

We examined the effect of HT on skeletal muscle autophagy in C2C12 myotubes. Autophagy can be broken down into four general stages: initiation, vesicle formation, fusion, and degradation. Initiation of autophagy is governed, by some degree, through its inhibition by mTOR, or activation by AMPK. Analysis of mTOR activation after 1 h of 40°C HT, through the levels of phosphorylation at Ser2448, showed no significant changes (Figure 2). Whereas, the phosphorylation of AMPK at Thr172, which is required for AMPK activation, was significantly elevated at 0 h recovery compared to TN and all other timepoints (all *p* < 0.05) (Figure 2). The main protein involved in autophagy initiation, ULK1, is regulated by many phosphorylation sites, but two main sites are: Ser757 (phosphorylated by mTOR to inhibit autophagy) and Ser555 (phosphorylated by AMPK to activate autophagy). The phosphorylation of ULK1 at Ser757 (*p* = 0.45) and Ser555 (*p* = 0.10) did not differ significantly across conditions (Figure 2).

### Regulation of Autophagy Nucleation and Elongation Proteins by 1 h 40°C HT

**FIGURE 3:**
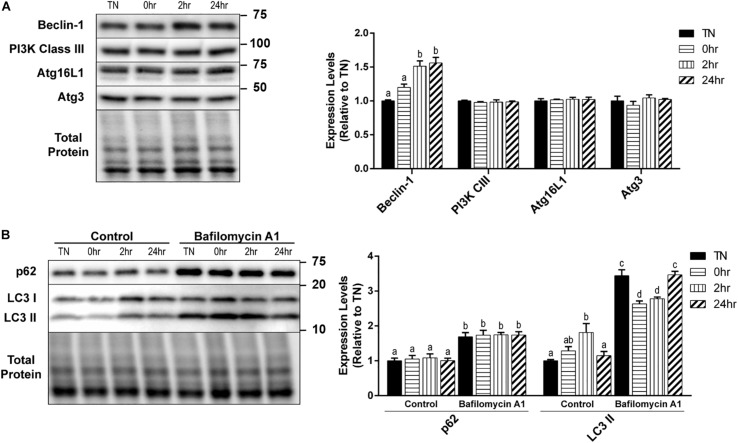
One hour 40°C HT regulation of autophagy proteins. C2C12 myotubes were maintained at 37°C, TN, or heated at 40°C for 1 h, and harvested immediately (0 h), or allowed to recover for 2 h, or 24 h. **(A)** Protein lysates of TN and heat treated myotubes were analyzed by Western blot for the key autophagy proteins: Beclin-1, PI3K Class III, Atg16L1, and Atg3. **(B)** A subset of myotubes were exposed to 3 h of Bafilomycin A1 (100 nM; BafA1) treatment to inhibit autophagy. Western blot analysis was conducted for autophagosome markers: LC3 II and p62. Western blot images and quantification are shown for all proteins. Bars that do not share similar letters denote statistical significance, *p* < 0.05 using a one-way or two-way ANOVA. Significant main effects of BafA1, HT, and interaction are displayed. Values are means ± SEM in relation to TN control.

Autophagy is a very complex pathway with a large variety of proteins that are involved in its activity. We further examined several key autophagy proteins that are vital to the process of vesicle formation and elongation. Unlike the alterations seen in the initiation and autophagosome markers, PI3K Class III, Atg16L1, and Atg3 expressions did not change with HT (Figure 3A). However, there was a significant increase in Beclin-1 expression at 2hr (*p* = 0.0016) and 24hr (*p* = 0.0009) recovery, compared to TN (Figure 3A).

There was a significant increase in LC3 II levels with 2 h recovery compared to TN (*p* = 0.0100), which returned to TN levels at 24 h recovery (*p* = 0.0454) (Figure 3B). However, HT did not have any effect on the levels of p62 (Figure 3B). The inhibition of autophagy by the addition of BafA1 lead to a significant increase in both LC3 II (*p* < 0.0001) and p62 (*p* < 0.0001) levels, compared to control (Figure 3B). Interestingly, in BafA1 treated cells there was a significant reduction in LC3 II levels in the 0 h (*p* = 0.0107) and 2 h (*p* = 0.0474) recovery compared to TN (Figure 3B). There was only a main effect with BafA1 treatment for p62 (*p* < 0.0001), while LC3 II had a main effect of BafA1 treatment (*p* < 0.0001), a trend for a main effect in HT (*p* = 0.0646), and a significant interaction effect (*p* < 0.0001).

### 1 h 40°C HT Triggers the Heat Shock Response

**FIGURE 4:**
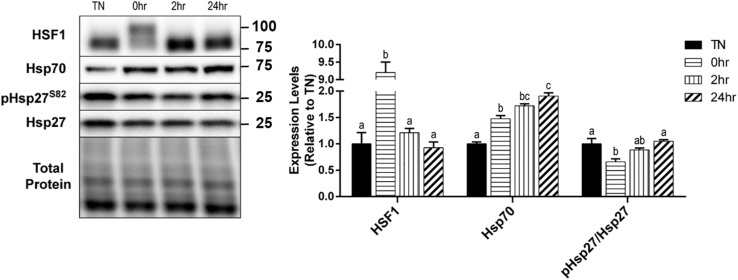
Heat shock protein response to 40°C HT. C2C12 myotubes were left at 37°C, TN, or exposed to 40°C for 1 h, HT, with cell harvest occurring immediately (0 h), or after 2 h, or 24 h of recovery. Protein lysates of TN and heat treated myotubes were analyzed by western blot for heat shock response proteins (HSF1, Hsp70, and pHsp27^S82^). Western blots and the quantifications are shown for all proteins. Bars that do not share similar letters denote statistical significance, *p* < 0.05 using a one-way ANOVA. Values are means ± SEM, expressed relative to TN.

Upon its activation, HSF1 becomes hyperphosphorylated, trimerizes, and relocates to the nucleus for transcriptional activation of key heat shock proteins. The hyperphosphorylation of HSF1 causes a shift in the appearance of the protein band’s molecular weight, when analyzed by SDS-PAGE and Western blot. To obtain a quantification of the shift in HSF1, images were analyzed for two bands, and are expressed as a fold-shift in the hyperphosphorylation to normophosphorylation ratio (Figure 4). When examined, HSF1 was significantly hyperphosphorylated at 0 h recovery compared to all other conditions (*p* < 0.0001) (Figure 4). Heat causes an elevation in Hsp70 levels and an elevation of phosphorylated Hsp27 at Ser86. Hsp70 expression was significantly elevated by HT at 0 h (*p* = 0.0081), 2 h (*p* = 0.0017), and 24 h (*p* = 0.0007) recovery, compared to TN (Figure 4). The phosphorylation of the small heat shock protein Hsp27, at Ser86, was transiently reduced at 0 h recovery compared to TN (*p* = 0.0194), that returned to TN levels by 24 h (Figure 4). The total Hsp27 protein levels did not change significantly with HT (*p* > 0.1).

### Regulation of Cell Stress Markers After Heating for 1 h at 40°C

**FIGURE 5:**
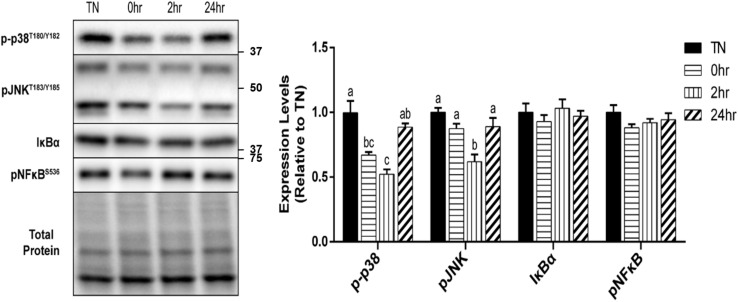
Cell stress marker expression levels after 1 h 40°C HT. C2C12 myotubes remained at 37°C, TN, or exposed to 40°C for 1 h, HT, with cell harvest occurring immediately (0 h), 2 h, or 24 h post-heating. Protein lysates of TN and heat treated myotubes were analyzed by western blot for cell stress markers: p-p38^T180/Y182^, pJNK^T183/Y185^, IκBα, and pNFκB^S536^. Western blots and the quantifications are shown for all proteins. Bars that do not share similar letters denote statistical significance, *p* < 0.05 using a one-way ANOVA. Values are means ± SEM in relation to TN.

In addition to the HSR, the effect of 1 h of 40°C HT on the expression of several cell stress markers were examined. The phosphorylation of the MAP kinase, p38, at Thr180/Tyr182, was significantly reduced by HT at 0 h (*p* = 0.0096) and 2 h (*p* = 0.0009) recovery compared to TN, but returned to TN levels by 24 h recovery (Figure 5). A similar, but delayed reduction in the phosphorylation of the stress kinase JNK at Thr183/Tyr185, was observed 2 h into recovery (*p* = 0.0030). Though there was a significant decrease in both the p38 and JNK MAPK’s, inflammatory stress markers, IκBα, and pNFκB at Ser536, were unaltered by HT (Figure 5).

## Discussion

Heat treatment can provide many benefits to skeletal muscle, such as reduced oxidative stress, improved insulin signaling, and increased mitochondrial biogenesis (Gupte et al., 2009; Liu and Brooks, 2012). Acute heating of C2C12 skeletal muscle cells at 40°C for 1 h has previously been shown to increase the phosphorylation of AMPK at Thr172, leading to transcription of PGC-1α, and activation of mitochondrial biogenesis (Liu and Brooks, 2012). In agreement, our data suggest that acute HT of 1 h upregulated autophagy initiation signaling, reflected by changes in the phosphorylation of AMPK at Thr172. Additionally, as expected, HT activated the heat shock response. These results support our hypothesis that a single bout of mild heat stress for 1 h can stimulate autophagy initiation signaling in addition to the heat shock response, in skeletal muscle cells.

Skeletal muscle requires efficient autophagic flux to allow for proper nutrient recycling and the removal of protein aggregates or dysfunctional organelles (Neel et al., 2013). It is well established that mTOR and AMPK are opposing regulators of ULK1, the main protein attributed to autophagy initiation is ULK1 (Kim et al., 2011; Russell et al., 2013). In the current study, we did not observe a HT effect on phosphorylation levels of mTOR at Ser2448, which is indicative of its activation. It has been suggested that phosphorylation of ULK1 at Ser555 levels may be a more efficient indicator of ULK1 activity, than levels of phosphorylation at Ser757 (Laker et al., 2017). With prolonged heat stress (HS) of 12 h, the phosphorylation of AMPK at Thr172 and ULK1 at Ser555 were significantly elevated in porcine skeletal muscle, but no changes were seen in the phosphorylation of ULK1 at Ser757 (Ganesan et al., 2017a). Here we show a similar response with a much shorter bout of heat, and a tendency (*p* = 0.10) for an increase in p-ULK1 at Ser555, with a large effect size (Cohen’s *f* = 0.41). Similarly, the ratio of ULK1 Ser555 to Ser757, an indicator of its activation state, tended to increase (*p* = 0.08), and had a large effect size (Cohen’s *f* = 0.59). These initiation events resemble those seen with exercise (Laker et al., 2017). Cells exposed to Bafilomycin A1 maintained the effects of HT on phosphorylated AMPK (*p* < 0.05; data not shown), further supporting changes in an initial step toward autophagy initiation by HT.

Here, we show that 1 h of HT significantly increased levels of Beclin-1 and LC3 II after 2 h of recovery. As expected, changes in LC3 II occurred sequentially after the elevation in autophagic initiation markers. Despite heat-induced changes in LC3 II, no effects on p62 were observed, suggesting an increase in overall autophagic flux or enhanced degradation. The ratio of p62/Beclin-1 has been suggested as another indicator of autophagic turnover (Klionsky et al., 2016). Our data support a time-dependent heat effect of reduced p62/Beclin-1 (*p* = 0.03), suggestive of turnover. This is in contrast to prolonged HS, in which both LC3 II and p62 were elevated simultaneously, signifying that degradation was inhibited, causing a buildup of autophagosomes in skeletal muscle (Ganesan et al., 2017a).

Using BafA1 to chemically inhibit autophagasome degradation, we observed that 1 h of HT decreased the BafA1-induced increase in LC3 II immediately after HT, until at least 2 h of recovery. It is possible that this short duration of heat is increasing initiation signals, without inducing high levels of cellular stress that would further drive autophagic flux, as supported by reduction in p38 and JNK phosphorylation, and no change in NF-κB signaling. Our finding is consistent with the effects of both muscle contraction and exercise to reduce autophagasome accumulation in the presence of chemical inhibition of autophagy, despite increased basal levels of autophagy (Jiang et al., 2014; Parousis et al., 2018). This effect is speculated to reflect a normalization of autophagic degradation, although the mechanism underlying this effect remains unclear.

Many of the proteins involved in autophagasome nucleation and elongation, such as Atg3, Atg16L1, and PI3K Class III, were unaffected by HT. Despite important roles in this process, these proteins are unlikely to be consumed during autophagy or be a rate-limiting factor in autophagic activity. However, 1 h of HT significantly increased Beclin-1 by 2 h of recovery, lasting until at least 24 h after HT. When Beclin-1 is bound to Bcl-2, the pro-autophagy activity of Beclin-1 is inhibited (He et al., 2012). The rise in Beclin-1 could be due to an increased need for unbound Beclin-1, to further propagate autophagy signaling. However, in porcine skeletal muscle exposed to 12 h of HS, PI3K Class III and Atg3 were significantly elevated compared to TN (Ganesan et al., 2017a). This may suggest that the proteins involved in autophagy nucleation and elongation may only be altered by a greater degree of stress, imposed by a longer duration of heat.

HSPs have many cellular functions, such as folding nascent or unfolded proteins, preventing protein aggregation, and degrading proteins (Bozaykut et al., 2014). The acute, transient hyperphosphorylation of HSF1, immediately after heating, signifies the activation of its transcription factor activity (Xia and Voellmy, 1997). We have previously seen that 15 min of recovery was enough for HSF1 hyperphosphorylation to return to thermoneutral (TN) levels (data not shown). An *in vivo* study reported no changes in skeletal muscle Hsp70 expression when pigs were exposed to 2, 4, or 6 h of HS (Ganesan et al., 2017b). Whereas we observed a significant duration-dependent elevation of Hsp70 during recovery. This is likely due, in part, to the lag in time between HSF1 activation of HSP transcription and the translation of Hsp70.

In contrast to the literature (Landry et al., 1992) our data demonstrate that the phosphorylation status of Hsp27, at Ser86, was acutely reduced by heat, which is known to alter its cellular function. When phosphorylated, Hsp27 is associated with cytoskeleton stabilization. Unphosphorylated Hsp27 is thought to form large oligomers, and play a role in anti-oxidant and chaperone activity, in addition to inhibition of apoptosis (Katsogiannou et al., 2014). This raises the possibility for the potential of heat to alter chaperone-mediated autophagy through this mechanism, which warrants further investigation.

Hsp27 can be phosphorylated by several kinases, including the MAP-kinases and PKCs (Marais et al., 2005), and appear to be most influenced by p38 MAPK (Larsen et al., 1997; Concannon et al., 2003). Unlike during exercise, in which mechanical stimulation increases p-p38 and subsequently increases p-Hsp27 (Hoffman et al., 2017), heat transiently suppressed the phosphorylation of p38, which may explain the reduced p-Hsp27 observed as this same time point. In addition to the altered HSP signaling, changes in MAPK signaling have been implicated in autophagy regulation (Sui et al., 2014). Interestingly, p38 MAPK has been suggested as both a negative and positive regulator of autophagy (Webber, 2010). Specifically, under conditions of starvation, an increase in p38 inhibits autophagy, whereas p38 depletion leads to an increase in LC3 (Webber, 2010). We observed a similar inverse pattern, in which the increase in LC3 II corresponded with reduced p-p38. In contrast, JNK activations is thought to promote autophagy, and thus does not appear to be a major factor responsible for heat-induced autophagy.

A standardized exposure to the autophagy inhibitor BafA1 (3 hr) was used to gain insight into autophagy flux, but resulted in varying degrees of autophagy inhibition occurring prior to (0 h and 2 h) or following heat exposure (24 h). An alternative approach of inhibiting autophagy for a consistent duration prior to the exposure to heat would result in varying degrees of autophagy inhibition, assay saturation, and durations exceeding recommendations (Klionsky et al., 2016). The data presented here are limited to heat-induced changes in the expression of autophagy-related proteins via Western blot, and are suggestive that the well-established nutrient sensor, AMPK, likely contributes to an upregulation in autophagy signaling in response to heat. The observed reduction in p-p38 may also contribute to autophagy. However, factors such as ER stress, oxidative stress, and lysosomal acidification and function may also be altered in response to heat. The role of these cell signaling changes on heat-induced autophagy cannot be ruled out. Future, mechanistic studies, incorporating immunocytochemistry of LC3 puncta and localization will be important to confirm and extend our findings.

## Conclusion

**FIGURE 6:**
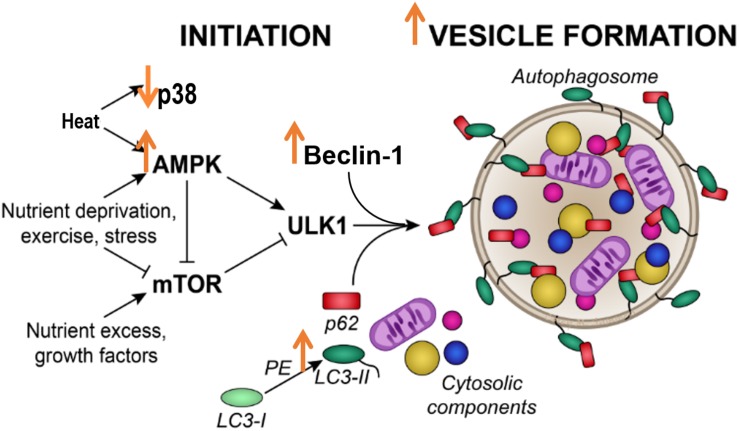
Acute heat exposure and autophagy. Heat acutely activates AMPK signaling, increasing Beclin-1 and LC3 II, indicators of autophagosome formation. Effects of heat are indicated by large orange arrows.

In conclusion, our study showed that, in C2C12 skeletal muscle cells, a single 1 h bout of 40°C HT increased autophagy initiation signals, and indicators of autophagosomal accumulation, along with activation of the heat shock response (Figure 6). Our data stem from cell culture experiments, are limited to healthy cells, and lack the systemic response to heat that would likely impact *in vivo* cellular metabolism. However, these results suggest that autophagy is altered by HT in skeletal muscle cells, and suggest that HT could be a therapeutic approach to target autophagy in skeletal muscle. Strategies such as hot water emersion, sauna, or localized heat application may provide therapeutic benefit, particularly in conditions with dysfunctional autophagy. Further studies are needed to directly investigate the potential difference in autophagic responses to varying heat loads and whether the activation of autophagy by heat can prevent or improve skeletal muscle pathologies.

## Data Availability Statement

The raw data supporting the conclusions of this article will be made available by the authors, without undue reservation, to any qualified researcher.

## Author Contributions

CS and RV were responsible for the conception and design of the study, completed the data collection, and were responsible for data analysis and drafting the manuscript. Both authors approved the final version of the manuscript.

## Conflict of Interest

The authors declare that the research was conducted in the absence of any commercial or financial relationships that could be construed as a potential conflict of interest.
