# Supplementary figures and images for: Acute Heat Exposure Alters Autophagy Signaling in C2C12 Myotubes
Source: Front Physiol. 2020 Jan 8;10:1521. doi: 10.3389/fphys.2019.01521 (PMC6960406; doi:10.3389/fphys.2019.01521)

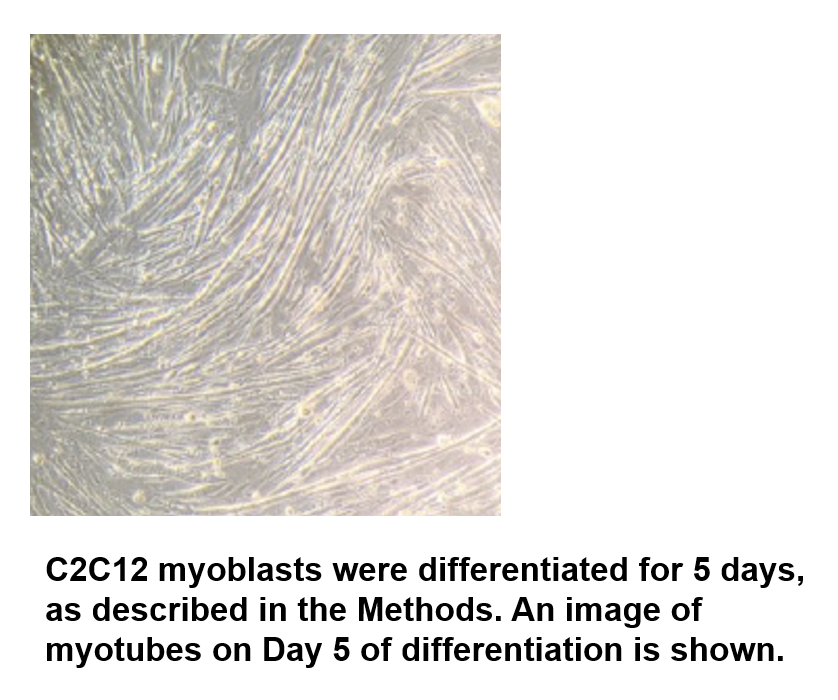

Supplement: Supplementary file 1 [file Image_1.TIF]
